# The mid-term outcome of total ankle arthroplasty and ankle fusion in rheumatoid arthritis: a systematic review

**DOI:** 10.1186/1471-2474-14-306

**Published:** 2013-10-26

**Authors:** Jacqueline van Heiningen, Thea PM Vliet Vlieland, Huub JL van der Heide

**Affiliations:** 1 Department of Orthopaedics J11-R74, Leiden University Medical Centre, P.O. box 9600, 2300 RC, Leiden, The Netherlands

**Keywords:** Rheumatoid arthritis, Ankle joint / talocrural joint, Three component mobile bearing prosthesis / third generation total ankle implant, Ankle arthrodesis / ankle fusion, Clinical outcome score

## Abstract

**Background:**

While arthrodesis is the standard treatment of a severely arthritic ankle joint, total ankle arthroplasty has become a popular alternative. This review provides clinical outcomes and complications of both interventions in patients with rheumatoid arthritis.

**Methods:**

Studies were obtained from Pubmed, Embase and Web of Science (January 1980 – June 2011) and additional manual search. Inclusion criteria: original clinical study, > 5 rheumatoid arthritis (population), internal fixation arthrodesis or three-component mobile bearing prosthesis (intervention), ankle scoring system (outcome). The clinical outcome score, complication- and failure rates were extracted and the methodological quality of the studies was analysed.

**Results:**

17 observational studies of 868 citations were included. The effect size concerning total ankle arthroplasty ranged between 1.9 and 6.0, for arthrodesis the effect sizes were 4.0 and 4.7. Reoperation due to implant failure or reoperation due to non-union, was 11% and 12% for respectively total ankle arthroplasty and arthrodesis. The methodological quality of the studies was low (mean 6.4 out of a maximum of 14 points) and was lower for arthrodesis (mean 4.8) as compared to arthroplasty (mean 7.8) (p = 0.04).

**Conclusions:**

17 observational and no (randomized) controlled clinical trials are published on the effectiveness of arthroplasty or arthrodesis of the ankle in rheumatoid arthritis. Regardless of the methodological limitations it can be concluded that both interventions show clinical improvement and in line with current literature neither procedure is superior to the other.

## Background

Ankle arthritis usually occurs in the later stages of rheumatoid arthritis (RA). In end stage ankle arthritis surgical management is often necessary, consisting of either arthrodesis or arthroplasty.

Arthrodesis (or ankle fusion) has long been considered to be the gold standard. The advantage of arthrodesis is a potential gain of walking ability due to relief of pain and decrease of deformity. However the loss of ankle motion, imposes stress on other joints and may, especially in RA patients, increase degenerative lesions in the mid- and forefoot [1].

Total ankle arthroplasty (TAA) preserves ankle range of motion (ROM), thereby compromising gait pattern to a lesser extent and imposing less stress on other joints [2,3]. Despite the potential advantages total ankle arthroplasty may not be the best treatment option for all patients as an implant requires adequate bone support and there is a risk of reoperation due to loosening [4].

The effectiveness of both interventions with mixed indications has been described in three systematic reviews, including second- and third generation implants and arthrodesis [5-7].

With respect to ankle fusion, the meta-analysis of Haddad et al., including thirty-nine original studies, showed that 73% (95% confidence interval (95% CI): 61–84), of all patients experienced a good result [5]. Concerning total ankle arthroplasty this meta-analysis, including ten primary studies, evaluated only second generation implants, and concluded that 78% (95% CI: 62–95) showed good results [5]. With respect to third generation implants Stengel et al., included ten studies and presented a weighted average improvement of 45.2 points (maximum score: 100 points) [6]. Gougoulias et al., reviewing thirteen studies and focusing on clinical failure- and survival rates of both second and third generation implants, showed 9.8% (95% CI 3.1– 16.5) implants failure after 5 years [7].

For both ankle arthrodesis and total ankle arthroplasty there has been no systematic review which included only RA patients or reported specifically the results of this patient group. Furthermore the systematic review, including arthrodesis studies, focused only on the clinical outcome scores whereas, complication types- and rates were not evaluated [5]. For outcome studies it is important to include and evaluate each diagnostic group separately, as factors as morbidity status are important to determine success [3]. Data of Stengel et al. showed that RA patients, overall have lower scores using functional scoring systems than patients with a single joint problem [6].

Therefore the aim of this study is to systematically review the literature regarding the effectiveness and safety of ankle arthrodesis and total ankle arthroplasty in RA patients. To enable comparisons among studies this review focuses on the standard surgical methods, i.e. isolated fusion of the talocrural joint by internal fixation methods and arthroplasty with third generation implant designs.

## Methods

### Search strategy

An electronic database search was performed from January 1, 1980 until June 14, 2011 by one author (JvH) in cooperation with a trained medical librarian. For the PubMed, Embase and Web of Science database the following search strategies were used:

Pubmed algorithm

• Ankle joint prosthesis OR arthroplasty OR arthrodesis OR internal fixator rheumatoid arthritis

• Search string for joint: ankle[MeSH Terms] OR ankle[All Fields] OR ankle joint[MeSH Terms]

• Search string for intervention: arthroplasty, replacement[MeSH Terms] OR arthroplasty[All Fields] OR Joint Prosthesis[Mesh] OR Joint Prosthesis[all fields] OR Joint Prostheses[all fields] OR replacement[all fields] OR arthrodesis[MeSH Terms] OR arthrodesis[All Fields] OR arthrodeses[All Fields] OR Internal Fixators[Mesh] OR Internal Fixator[all fields] OR Internal Fixators[all fields] OR fusion[all fields]

• Search string for diagnosis: arthritis, rheumatoid[MeSH Terms] OR rheumatoid[All Fields] AND arthritis[All Fields]

Embase algorithm

• (Ankle/ or ankle.mp.) AND (exp Arthroplasty/ OR arthroplasty.mp. OR joint prosthes*.mp. or Joint Prosthesis/ OR replacement.mp. OR exp arthrodesis/ OR arthrodes*.mp. OR internal fixator*.mp. or Internal Fixator/)

Web of Science algorithm

• (rheumatoid arthritis.mp. or Rheumatoid Arthritis/)

Regarding the limited reviewed literature concerning this topic, a Cochrane database search was not performed. To search for potential additional studies the electronic database search was supplemented by a manual check of references of recent reviews and primary full text articles identified with the search strategy, as described above.

### Selection of articles

All titles, abstracts and selected full text articles were screened by two authors independently (JvH and HvdH). The review sought randomized controlled trials (RCTs), controlled clinical trials and observational studies in which only RA patients were described or in which data from RA patients could be extracted from the general data, and were written in English. Articles published before 1980 were excluded.

Titles and abstract were screened using three general criteria:

• Original clinical study (no reviews or case reports)

• The intervention(s) evaluated was arthrodesis, total ankle arthroplasty or both

• The efficacy of interventions was tested

In case of a potentially relevant title and abstract full text articles were examined using the aforementioned inclusion criteria plus the following criteria:

• Studies that reported on at least five RA patients with end-staged rheumatic ankle(s) were included (the cut-off point of 5 was chosen as there are very few studies including large numbers of RA patients).

• The intervention concerned internal fixation arthrodesis of the talocrural joint or arthroplasty with a third generation, three-component mobile bearing prosthesis. If studies presented the outcome of a combination of internal and external fixation arthrodesis or second and third generation implants in arthroplasty, data derived from internal fixation or third generation implants could be extracted separately.

• The clinical outcome had to be evaluated by using an ankle scoring system, designed for evaluating surgical interventions for ankle problems, including the Kofoed [8], Amercian Orthopaedic Foot and Ankle Society (AOFAS) ankle- and hindfoot score [9], Foot Functional Index (FFI) [10] and Mazur score [11]. All scoring systems use somewhat similar items as pain, function, range of motion (ROM), and deformity and all have a domain of 100 points [12]. The outcome had to be measured at least once postoperatively.

Exclusion criterion:

• Studies on the effectiveness of arthrodesis after a failed total ankle arthroplasty were excluded.

### Data extraction

The study characteristics and clinical outcomes were extracted from the selected full-text articles. For continuous data, preferably the mean, range and standard deviation (SD) were extracted. If not presented in the study, the mean, range and SD data were calculated whenever possible.

Study characteristics included:

a. Year of publication, time period in which patients were examined, average duration of follow up (years), number of RA patients included in the study, percentage of RA patients in the total included patient population and gender distribution.

Clinical outcomes included:

a. Type of ankle scoring system, response rate (the total number of RA patients evaluated divided by the number of RA patients which were included) and postoperative and, if available, preoperative ankle scores (out of 100 points). Comparison between the various scoring systems is based on the overall outcome score, as individual items as pain, function and alignment were often not available. To enable comparison between Foot Functional Index (FFI) with other scoring systems, the FFI score was inverted by subtracting the score from 100. As an optimal clinical outcome, measured with the FFI is represented as 0.

b. Frequencies of common complications after ankle surgery: peri- and postoperative fractures and infection.

c. Failure rates: proportion of patients undergoing reoperation due to non-union in arthrodesis or implant removal in arthroplasty followed by implantation of new component(s) or fusion.

### Quality assessment

As a systematic review can be of great value in evidence based medicine, it is important that data, upon which the review is based, are reliable and obtained within a sound methodological design. As a gold standard for internal and external validity evaluation of observational intervention studies does not exists, the authors (JvH, HvdH and TVV) comprehended a user-friendly seven item rating system composed of a quality checklist for intervention studies and diagnostic tests [13,14], shown in Table 1. This quality appraisal system generally agrees with other appraisal tools upon most important, well-known, flaws as selection- and observation bias and confounding [15,16]. However with this system, discrimination is possible between the reporting- and methodological quality. To allocate the evaluation criteria to the four most important elements for conducting evidence: study design, subjects (patients), outcome and analysis, this tool can elucidate the flaws and strengths of the included studies.

**Table 1:** Descriptors of methodological quality assessment

**Evaluation criteria**	**Score**	**Descriptors**	
Study design	Patient evaluation on clinical relevant time points (3)	2	Patients were evaluated with the same criteria at more than one clinical relevant time point (pre- and post-operatively).
		1	Patients were evaluated at more than one time point (but the above criteria were not fulfilled).
		0	Patients were evaluated at only one point in time.
	Evaluation outcome measures (8)	2	Outcome measures were administered by an evaluator who was blind to treatment or self-report was considered as provided by an independent person.
		1	Evaluators were not blinded, but were not involved in treatment or self-report was administered by treatment providers.
		0	Outcome measures were obtained by unblended treatment providers.
Subject	Eligibility criteria (10)	2	Inclusion and exclusion criteria were defined and designed to yield a study group generalizable to clinical situation.
		1	Some available inclusion and exclusion information regarding included patients, but information prevents to generalize study results to a specific population.
		0	No information on inclusion and exclusion criteria and limited patients descriptors.
	Response rate (12)	2	90% or more of the patients enrolled or eligible for study were evaluated for outcomes.
		1	More than 70% of the patients eligible for study or enrolled were evaluated for outcomes.
		0	Less than 70% of patients eligible for study or enrolled were evaluated.
Outcome	Outcome definitions (16)	2	Primary outcome measure, which represented important clinical outcomes and appropriate psychometric properties (reliability, validity, responsiveness).
		1	Primary outcome measure was evident, but was insufficient in either its clinical relevance or its psychometric properties.
		0	Primary outcome was irrelevant or methodologically unsupported.
Analysis	Statistical analysis (21)	2	Authors conveyed both statistical significance and size of treatment effect. Indicated by p-values, confidence intervals, effect sizes, or other similar methods.
		1	Statistical significance of described means and p-values, but no confidence intervals/effect sizes.
		0	Descriptive, statistical information was not reported.
	Data collection (22)	2	Complete data collection or a strategy for handling missing data was documented or a specific analysis was conducted to determine the impact of missing data.
		1	Missing data was not an apparent issue but no strategy for handling missing data was described.
		0	Missing data may have been an issue and no strategy for missing data was documented.

*number between parentheses refers to descriptor of the methodological quality assessment system by Macdermid et al. [13].

Two authors (JvH and HvdH) independently assessed the methodological quality of each study included in this review. Each item received 0, 1 or 2 points (descriptors Table 1), so the maximum score of 14 points reflecting studies with a good methodological quality. Discrepancies were examined by a third observer (TVV) independently and consensus between the three authors was reached. As one of the raters is also the author of one of the included articles (HvdH), his study was also scored blinded by the third reviewer (TVV).

The GRADE criteria were used to describe the overall quality of evidence and strength of recommendations of this study.

### Data- and statistical analysis

Due to absence of preoperative scores, incomplete measures of variability, heterogeneity of the patient populations and variety among outcome scoring systems, formal pooling and subsequently a comprehensive statistical analysis was not possible. Therefore, descriptive statistical analyses were used. Results are expressed as mean and standard deviation (SD). Statistical analysis was performed using SPSS (IBM SPSS Statistics for Windows, Version 20.0).

For studies in which the pre- and postoperative clinical score were available we calculated the effect size by subtracting the pre-operative score from the postoperative score and divide this number by the standard deviation of the preoperative score. The methodological scores between the two different observers were compared with an intraclass correlation coefficient (ICC). The methodological scores between the two different treatment options were compared with a Student t-test. Correlation between methodological scores, impact factor of the journal and study size was calculated using a Spearman correlation coefficient.

## Results

### Search and selection of articles

**Figure 1:**
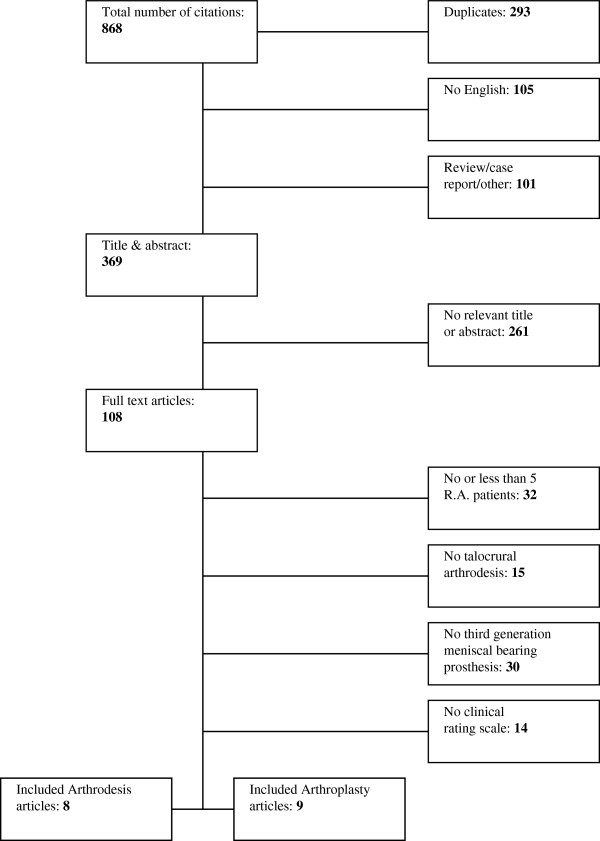
The article selection process.

The initial electronic databases search yielded a total of 868 titles. The process by which the final selection of articles was made is visualized in Figure 1. After excluding duplicates, articles not written in English and non-original clinical studies, 369 potentially relevant titles and abstracts were screened for intervention type (arthrodesis or arthroplasty) and the efficacy of intervention, finally 108 full text articles were examined. Consensus was reached by both reviewers (JvH and HvdH) on 17 full text articles.

### Description of included studies

Table 2 lists the characteristics of the studies incorporated in this review. Eight articles evaluated arthrodesis and nine reported the outcome of total ankle arthroplasty. The selected papers were published between 1989 and 2009.

**Table 2:** Demographic information of included studies

**Author**	**Centre**	**Year**	**Recruitment period**	**Follow up (yrs.)**		**n included RA patients**	**Age (yrs.)**		**Male**	**Manufacturer prosthesis (TAA) or operation technique (arthrodesis)**
				**Mean**	**Range or SD**		**Mean**	**Range or SD**		
**Total Ankle Arthroplasty (TAA)**										
van der Heide et al.	Radboud University, Nijmegen Medical Centre, The Netherlands	2009	1996 – 2004	2.7	1-9	54	55	27-82	19%	STAR & BP
Schutte et al.	Maartenskliniek, Nijmegen, The Netherlands	2008	1999-2004	2.3	1-5.6	29	57	37-81	34%	STAR
Nelissen et al.*	Leiden University Medical Centre, Leiden, The Netherlands	2006	2001-2003	2.0	SD: 0,4	15	61	SD 8,6	20%	BP
San Giovanni et al.	UHZ Sports Medicine Institute, Coral Gables, Florida	2006	1990-1997	8.3	5-12.2	23	61	28-79	9%	BP
Doets et al.	Slotervaart Hospital, Amsterdam, The Netherlands	2006	1988-1999	7.6	0.4-16.3	76	58	27-81	82%	LCS & BP
Carlsson et al.	Malmö University Hospital, Malmö, Sweden	2005	-	4.6	4-5	5	66	53-83	40%	STAR
Bonnin et al.	Clinique Sainte Anne Lumière, Lyon, France	2004	1997-2000	2.9	2-5.7	29	54	28-77	38%	SALTO
Anderson et al.	Malmö University Hospital, Malmö, Sweden	2003	1993-1999	4.3	3-6.6	22	61	27-75	33%	STAR
Wood et al.	Wrightington Hospital NHS Trust, Wigan, United Kingdom	2000	-	5.4	5-6	7	63	36-76	17%	STAR
Mean				4.5			59.5		32%	
Total						260				
**Arthrodesis**										
Anderson et al.	Malmö University Hospital, Malmö, Sweden	2005	1991-2002	7.0	2-12	51	56	24-76	17%	Percutaneous & open
Kennedy et al.	Hospital for special surgery, New York, New York	2003	1992-1996	3.8	2,1-6,3	20	56	31-81	24%	Open
Shinomiya et al.	School of Medicine University, Tokushima Japan	2003	1986-1996	-	-	17	52	σ 7,8	12%	-
De Palma et al.	Università di Ancona, Ancona, Italy	2000	1994-1995	3.0	2-5	7	48	35-64	0%	Arthroscopic
Lauge-Pedersen et al.	Lund University Hospital, Lund Sweden	1998	1993-1997	2.3	0,3-4,4	10	66	45-80	40%	Percutaneous
Stranks et al.	Queen Alexandra Hospital, Portsmouth, United Kingdom	1995	1989-1991	1.6	1-2,5	8	59	51-70	13%	Open
Moran et al.	Freeman Hospital, Newcastle, United Kingdom	1991	1977-1986	5.0	2-12	26	54	28-80	15%	Percutaneous & open
Sowa et al.	John Hopkins University, Baltimore, Maryland	1989	1980-1985	4.0	1,1-6,6	6	60	32-77	17%	Open
Mean				3.8			56.4		17%	
Total						145				

* = As the range was not available, the standard deviation is reported.

¯ = information has not been described within the original article.

*Abbreviations:STAR* Scandinavian Total Ankle Replacement, *BP* Buechel Pappas, *LCS* Low Contact Stress prosthesis.

The 17 included studies were performed by 14 different centres from Italy, Japan, The Netherlands, Sweden, United Kingdom and The United States. The studies were carried out between 1977 and 2004. The mean follow up time varied from 1.6 to 8.3 years. For arthrodesis the mean follow up period was 3.8 years, and 4.5 years for arthroplasty. Also the number of included RA patients showed variety (n = 5 to 76). In total, the 17 studies included 145 RA patients with talocrural arthrodesis and 260 RA patients with third generation total ankle prosthesis.

### Methodological quality

The methodical quality of the 17 included studies was assessed with the seven item rating system (Table 1). The intraclass correlation coefficient (ICC) between the two reviewers (JvH and HvdH) was 0.84 (95% CI 0.61-0.94).

As depicted in Table 3, the mean score of the arthroplasty studies was 7.8 (SD 2.2), which was substantially higher than 4.8 (SD 1.3) for the fusion studies (p = 0.04) (maximum score = 14). The correlation coefficients between the methodological quality score and the impact factor of the journal was 0.7 (p = 0.004) and for the methodological quality and the amount of patients in the study was 0.5 (p = 0.04). Furthermore the correlation coefficient of the methodological quality score and year of publication (before and after 2005) was 0.6 (p = 0.06). So, studies with a high quality score were published in higher impact journals, included more patients, and the quality of the studies increased over time.

**Table 3:** Methodological quality of included studies

**Author**	**Year**	**Study design**		**Subject**		**Outcome**	**Analysis**		**Total consensus**
		Patient evaluation on clinical relevant time points (3)*	**Evaluation outcome measures (8)**	**Eligibility criteria (10)**	**Response rate (12)**	**Outcome definitions (16)**	**Statistical analysis (21)**	**Data collection (22)**	**(0–14 points)**
**TAA**									
van der Heide et al.	2009	0	1	0	2	1	1	1	6
Schutte et al.	2008	2	1	1	2	2	1	1	10
Nelissen et al.	2006	2	0	1	2	2	1	1	9
San Giovanni et al.	2006	1	0	1	2	1	1	1	7
Doets et al.	2006	2	0	1	2	2	2	2	11
Carlsson et al.	2005	0	0	1	2	1	0	2	6
Bonnin et al.	2004	2	0	1	2	1	1	1	8
Anderson et al.	2003	2	1	1	2	1	1	1	9
Wood et al.	2000	0	0	0	2	1	0	1	4
Mean									7.8
**Arthrodesis**									
Anderson et al.	2005	0	1	1	0	1	0	2	5
Kennedy et al.	2003	0	0	1	1	1	0	1	4
Shinomiya et al.	2003	0	0	1	2	1	0	0	4
De Palma et al.	2000	2	0	0	2	1	0	1	6
Lauge-Pedersen et al.	1998	0	0	0	2	0	0	1	3
Stranks et al.	1995	0	0	1	2	1	0	1	5
Moran et al.	1991	0	1	1	2	1	0	2	7
Sowa et al.	1989	0	0	1	1	1	0	1	4
Mean									4.8

* = number between parentheses refers to descriptor of the methodological quality assessment system by Macdermid et al. [13].

0 = minimal 1 = intermediate and 2 = maximal score for methodological quality.

Definition of each individual score is presented in Table 1.

We analysed the outcome separately for all studies with a methodological score of 7 and above, but only one study in the fusion group had a methodological score of seven and this study didn’t describe the pre-operative score so it was not possible to analyse only the studies with of moderate to good methodological score.

As all included studies were observational studies they would be graded as “low evidence” according to the GRADE classification, furthermore, due to the low quality it should be downgraded and the grade of evidence assigned should be “very low”.

### Effects of intervention

In Table 4 clinical outcome scores are presented. Preoperative data and measures of variability were absent in 12 articles [17-28]. This restrains the possibility to measure the effect size and to compare the preoperative status of the two groups. In addition, non-standardized outcome descriptors such as satisfied (yes/no) or poor outcome (yes/no) were used in a considerable number of studies.

**Table 4:** Primary outcome: total ankle replacement or arthrodesis

**Author**	**Outcome**			**Pre-operative outcome score**			**Post-operative outcome**			**Increase score**	**Effect size**
	**n of evaluated RA patients**	**Lost to follow up**	**Type of ankle scoring system**	**Mean value**	**Mean SD**	**Range**	**Mean value**	**Mean SD**	**Range**		
**TAA**											
van der Heide et al.	52	0%	Kofoed	-			73.0	16.0	21-92		
Schutte et al.	29	0%	FFI	40.0	14.0		66.0	19.0	-	26.0	1.9
	29		Kofoed	-			69.0	20.0	-		
Nelissen et al.	15	0%	AOFAS	22.0	9.7		80.0	8.0	-	58.0	6.0
San Giovanni et al.	21	0%	AOFAS	-			81.0	-	40-92		
Doets et al.*	76	0%	LCS	36.1	12.5	34-39	81.5	13.1	78-84	45.4	3.6
	76		AOFAS	26.5	11.6	24-29	77.7	14.0	75-81	51.2	4.4
	76		Kofoed	26.9	13.5	24-30	74.0	14.9	71-77	47.1	3.5
Carlsson et al.	5	0%	AOFAS	-			82.2	7.2	74-92		
Bonnin et al.	29	1%	AOFAS	-			84.2	14.0	-		
Anderson et al.°	22	0%	Kofoed	35.0		21-43	71.0	-	21-96	36.0	
	22		AOFAS	-			78.0	-	33-100		
	22		Mazur	-			72.0	-	28-91		
Wood et al.	7		Kofoed	-			74.0	-	-		
Mean				31.1	12.9		76.0	14.0		44.0	3.9
**Arthrodesis**											
Anderson et al.¯	35	2%	Mazur max 90 points	-			61.3	12.5	23-73		
	35		AOFAS max 86 points	-			64.9	14.8	15-85		
Kennedy et al.	17	15%	Mazur	24.0	-	2-30	69.0	-	5-83	45.0	
	17		Moran	28.0	-	2-32	79.0	-	17-89	51.0	
Shinomiya et al.^+^	17	-	JOA score	-			66.8	4.1	-		
De Palma et al.	7	0%	AOFAS	26.7	13.0	14-45	79.0	10.3	67-94	52.3	4.0
Lauge-Pedersen et al.	9	0%	Mazur	-			63.9	10.8	50-85		
	9		Moran	-			88.8	8.5	73-97		
Stranks et al.	8	0%	Mazur	-			58.3	16.2	32-80		
Moran et al.	21	0%	Mazur	-			61.7	7.7	48-76		
Sowa et al.	6		Mazur	16.8	13.6	8-43	80.3	13.1	65-95	63.5	4.7
Mean				23.9	13.3		70.3	10.9		53.0	4.4

* = Post-operative score was after 1 year evaluation.

° = The values are given as the median, with the range in parentheses.

¯ = Both outcome scores are adjusted to different max. score.

^+^*=* Arthrodesis patients served only as control group for reviewed second generation total ankle implant.

*Abbreviations:AOFAS* American Orthopaedic Foot & Ankle Society, *FFI* Foot Functional Index, *JOA* Japanese Orthopaedic Association.

The AOFAS, Mazur and Kofoed score were the most frequently reported evaluation tools. As the range of motion (ROM) should be zero after a successful fusion, this is not an appropriate tool for evaluating the success of arthrodesis. Except for the study of Anderson which mentioned an adjusted clinical outcome score; the ROM was not taken into account [11,24]. Shinomiya et al. explicitly mentioned the inclusion of ROM [20].

As formal pooling was not possible, only a descriptive analysis was performed to present the data. The preoperative score for an implant ranged from 22.0 to 40.0 points (SD range: 9.7 - 14.0). In the arthrodesis group this score ranged from 16.8 to 28.0 points (SD range: 13.0 - 13.6). The postoperative score for total ankle arthroplasty ranged from 66.0 to 84.2 points (SD range: 7.2 – 20.0), and for arthrodesis this was between 58.3 and 88.8 points (SD range: 4.1–16.2). The effect size could only be determined in 5 studies. The effect size for an implant ranged from 1.9 to 6.0 and in the arthrodesis group the original studies presented effect sizes of 4.0 and 4.7.

### Complications

Table 5 lists the complication- and failure rates per intervention type. The most frequently reported type of complication of arthroplasty was a perioperative fracture (26%). Patients who underwent fusion experienced mainly wound healing problems (17%). The failure rate was 11% for arthroplasty and 12% for arthrodesis.

**Table 5:** Complications: total ankle replacement or arthrodesis

**Author**	**n of ankles**	**Complications**				**Failure**
		**Perioperative fracture (n)**	**Wound healing problems (n)**	**Postoperative fracture (n)**	**Deep infection (n)**	**Reoperation due to implant removal (TAA) or non-union (arthrodesis)(n)**
**TAA**						
van der Heide et al.	56	29% (16)	5% (3)	-	4% (2)	9% (5)
Schutte et al.*	29	-	-	-	-	-
Nelissen et al.	15	-	7% (1)	13% (2)	-	0%
San Giovanni et al.	31	32% (10)	13% (4)	13% (4)	0% (0)	6% (2)
Doets et al.	93	29% (27)	4% (9)	10% (4)	4% (4)	16% (15)
Carlsson et al.*	5	-	-	-	-	0%
Bonnin et al.*	29	7% (2)	-	-	-	0%
Anderson et al.	28	-	9% (2)	-	-	24% (6)
Wood et al.	7	14% (1)	14% (1)	-	-	0%
Total	293	26% (56)	9% (20)	7% (10)	3% (6)	11% (28)
**Arthrodesis**						
Anderson et al.	35	-	9% (3)	3% (1)	3% (1)	14% (5)
Kennedy et al.	20	-	10% (2)	-	5% (1)	15% (3)
Shinomiya et al.	17	-	-	-	-	-
De Palma et al.	7	0%	0%	0%	0%	0%
Lauge-Pedersen et al.	11	0%	0%	18% (2)	0%	0%
Stranks et al.	8	-	-	-	-	0%
Moran et al.	30	-	40% (12)	7% (2)	10% (3)	20% (6)
Sowa et al.	6		17% (1)	17% (1)	0%	0%
Total	134	0%	17%	7%	6%	12%

* = complications/failure in RA patients was not described.

Type of complication not described or not possible to extract from published data.

**Figure 2:**
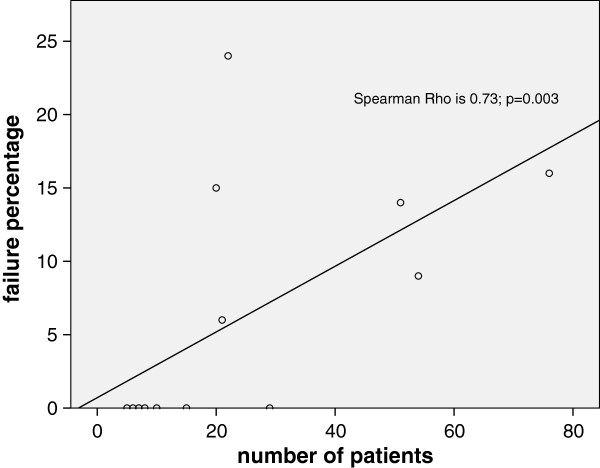
Correlation between study size and number of reported failures.

Regarding numbers of complications and failure there is a wide range among different studies. Doets et al. reported 29% perioperative fractures, as Anderson et al. did not report any fractures [2,23]. Furthermore studies which included a small number of patients e.g. De Palma et al. did not report failure. However studies with > 20 patients did [29]. Overall we find heterogeneity in the amount of complications. Figure 2 visualizes a positive correlation Rho = 0.73 (p = 0.003) between study size and number of reported failure rate. When larger amounts of patients were included, higher failure rates were reported. Due to publication bias it is possible that only smaller series with good results were published. Whereas series with high complication- and, or failure rates will not be published.

## Discussion

Neither randomized controlled trials nor controlled clinical trials are published on the effectiveness of arthroplasty or arthrodesis of the ankle in rheumatoid arthritis. This review included 17 observational studies, all with low methodological quality. Furthermore due to clinical heterogeneity, formal pooling was not possible and because the absence of preoperative scores and measures of variability, statistical analyses were limited. These limitations result in a systematic review with low quality of evidence according to the GRADE guidelines [30].

Both interventions show a comparable clinical improvement. For arthrodesis the postoperative scores, with a mean follow up time of 3.8 years, ranged from 58.3 to 88.8 (maximum of 100) and for total ankle arthroplasty with third generation prostheses, with a mean follow up time of 4.5 years, the scores ranged between 66.0 and 84.2. The effect sizes for arthrodesis were 4.0 and 4.7, and the effect size in the implant group ranged between 1.9 and 6.0. However when interpreting the effect sizes for arthrodesis, the non-adjustment of clinical outcome score, regarding the ROM, must be bear in mind. A successful fusion will obviously result in no range of motion. This could imply underestimation up to 10%, of the result of arthrodesis. Moreover the failure rates, in this review characterized as reoperation due to implant removal in the arthroplasty group or to non-union in arthrodesis, were similar (11–12%). Expect for a study by de Palma et al., who included only 7 patients, we obtained just results of open ankle fusion techniques. Although arthroscopic procedures show good results by experienced surgeons, this technique seems not suitable to correct large deformities which are often present in RA patients [29,31]. In future research the value of arthroscopic fusion in this specific patient group could be investigated.

The clinical outcome for both interventions in RA patients, presented in this review, is overall consistent with earlier published results in a mixed population by Stengel et al. and Gougoulias et al. [6,7]. At moment of writing only Haddad et al. as well systematically reviewed both arthroplasty and arthrodesis; however the authors focused only on the second generation implants. Nevertheless, comparable with our data, they found similar clinical outcomes for both surgical interventions [5]. To weigh each intervention, this review also focused on complication- and failure rates. Concerning arthrodesis we reported a non-union rate of 12%, which is consistent with earlier presented publications [5,32]. Complications as deep infections, loosening and fractures after implant surgery were only evaluated by Stengel et al. [6]. The failure rates for total arthroplasty were evaluated in two original studies [32,33] and the three aforementioned reviews [5-7]. The peri- and postoperative complications as superficial and deep infections (resp. 10.8% and 1.6%) are comparable to findings of this review (resp. 9% and 3%) [6]. Although failure of an implant was defined as implant removal and not as revision, the failure rate 11% (range: 0–24%) is also in line with the findings of Stengel et al., Gougoulias et al. and Saltzman et al. [6,7,33]. However the failure rate was higher compared to the second generation implants evaluated by Haddad et al., who showed revision rate of 7%, but was lower compared to a large observational study published in 2007 l [5,32]. Unfortunately the authors did not describe the type and or generation of implants, which hampers to explain discrepancies between their result and the overall low revision rate [5,32]. Soohoo et al. described a moderate revision rate for implants of 9% after one year, but after five years it was 23% compared to respectively 5% and 11% for arthrodesis [32]. This implicates that the risk of major revision surgery increases in the long-term. However this review could not demonstrate such a trend, though the reviewed arthroplasty studies showed a mean mid-term follow up time of 4.5 years (range: 2.0 - 8.3 years). Nevertheless, regarding the increased popularity of ankle implants, long-term results (> 5 years) and revision rates are needed.

The strength of this review lies in the assessment of the methodological quality of included studies. Furthermore we focused on patients with R.A., which is an unique patient population compared to patients with a monoarticular problem. Also inclusion of results of the newer third generation implants has not been extensively described before. An important weakness of this review is the outcome scoring system. Only studies, which applied a 100 point ankle scoring scale, were included. Scoring systems which provided nominal outcomes e.g. good and poor were excluded. This was done to obtain objectified data and to compare, in which extent this is possible, the different evaluation tools. Notwithstanding this limitation, the great diversity among (non-validated) scoring systems i.a. prevented pooling and therefore this review provides subsequently suboptimal evidence.

Regrettably three major issues concerning the included studies; 1) small study size, 2) clinical heterogeneity and 3) poor methodological design, restrained us to present a comprehensive statistical analysis with pooled data.

1) Small study size: 9 out of the 17 included studies included < 20 patients [17-19,25,28,29,34,35]. The low failure- and complication rate in small studies needs to be noted as this could be an indication for publication bias, which is important as published studies can have a high impact on daily medical practice.

2) Clinical heterogeneity: Neither pre-existing conditions, preoperative functional status e.g. the health assessment questionnaire (HAQ), nor all preoperative clinical outcome scores were presented. This prevented pooling, but might also lead to selection bias. Furthermore it is important to bear in mind that a negative and significant correlation between functional scores and the activity of the rheumatic disease can explain a low clinical outcome with similar or even better treatment effects in patients with a high disease activity [24].

3) Poor methodological design: In the included articles it concerned paucity of statistical parameters, heterogeneity in complication and failure denotations, as well as the usage of a great variety of non-validated and non-uniform outcome scores. However this review revealed the methodological quality of included studies no certain cut-off value was applied as exclusion criterion regarding the small number of studies (17). Statistical parameters as the standard deviation, which was absent in 4 studies [19,21,23,24,26] prevented measuring the effect size.

The heterogeneity in definitions of complication and failure prevented also comparison. Several authors characterized e.g. failure as revision but also as radiographic loosening. These different described endpoints raise the question whether the presented results are consistent with patients’ satisfaction.

The use of non-validated rating scales as mentioned before is also a problem, as the outcomes are not standardized and therefore not reproducible, their use hampers evaluation. Of the 17 included studies only Schutte et al. applied a validated rating scale, the FFI [36,37]. Furthermore, apart from Anderson et al., authors of the arthrodesis studies did not describe adjustment of the functional score to the normal loss of motion after fusion of the talocrural joint, which makes it a non-uniform scoring system [24]. As not all authors mentioned adjustment, we could not adjust the maximum score. Therefore, as stated earlier, the effect size must be interpreted with awareness of underestimation of successful fusion.

Even when all included studies would have been methodological well designed, indication bias remains a threat to validity in observational studies. Indication bias is controlled best by an RCT, however randomization and control is not always feasible. When two interventions have different profiles, both patients and surgeons have a preferable intervention. Strong preferences make recruitment difficult if not impossible [38]. To overcome the problems with randomization a clinical controlled trial can be preferable. Moreover as historically most advances in surgical knowledge have been accepted on the basis of non-randomized studies [38].

To improve the quality of research in the future, the research proposal should include and evaluate each diagnostic group separately, as factors as morbidity status are important to determine success [3]. To make studies comparable, the same validated rating scale, preferably a patient reported outcome measure would be valuable [36]. Currently the FFI, and recently also the Swedish version of the self-reported foot and ankle score (SEFAS) and the Manchester-Oxford Foot Questionnaire (MOFXFQ) are validated outcome measures [36,39,40]. With well-defined cohorts, outcomes, endpoints, exposures, predictors and possible confounders, a prospective observational study can give important contributions to ankle surgery in rheumatic patients.

## Conclusions

Currently no controlled clinical trials on the effectiveness of arthroplasty or arthrodesis of the ankle in rheumatoid arthritis have been published. Regardless of the methodological limitations it can be concluded from 17 observational studies, that both interventions show clinical improvement. This is in line with current literature that indicate that no procedure is superior to the other. However it remains to be established which treatment gives the best results in longer term. These interventions should preferably be studied with a randomized controlled trial, however cohort studies with sound methodological methods could also be of value.

## Competing interests

Each author certifies he/she has no commercial associations or non-financial competing interest that might pose a conflict. The authors did not receive grants or funding in support for preparation of this manuscript.

## Authors’ contributions

JvH & JM Langenhoff (medial librarian) carried out the electronic database search. Subsequently the selection of articles was performed by JvH & HvdH. For the quality assessment JvH & HvdH independently scored the included studies, discrepancies were examined by TVV after which consensus was reached. Data extraction was carried out by JvH. Statistical analysis was performed by HvdH. JvH drafted the manuscript. HvdH helped and commentated on the discussion. All authors read and approved the final manuscript.

## Pre-publication history

The pre-publication history for this paper can be accessed here:

http://www.biomedcentral.com/1471-2474/14/306/prepub
